# De-stress your physiological activation by compressing your imagination: a brief session of hypnosis decreases sympathetic stress response in moderately stressed dentists

**DOI:** 10.3389/fpsyg.2025.1577325

**Published:** 2025-06-20

**Authors:** Luca Queirolo, Enrico Facco, Andrea Roccon, Elisa Pistollato, Adolfo Di Fiore, Teresa Fazia, Christian Bacci, Gastone Zanette

**Affiliations:** ^1^Department of Neurosciences, School of Dentistry, University of Padua, Padua, Italy; ^2^Department of Philosophy, Sociology, Education and Applied Psychology, University of Padua, Padua, Italy; ^3^Ist. Franco Granone-Centro Italiano di Ipnosi Clinico Sperimentale, Turin, Italy; ^4^Department of Medicine, University of Padua, Padua, Italy; ^5^Department of Brain and Behavioral Sciences, University of Pavia, Pavia, Italy

**Keywords:** dentistry, stress, HRV, EDA, hypnosis, physiology

## Abstract

**Aim:**

This study aims to explore the effect of hypnosis on dentists’ physiological stress management.

**Methods:**

The study included 20 dentists (mean = 30, SD = 7.37) from the Dental Clinic of the University of Padua. Stress assessment was performed by recording several physiological parameters, including heart rate (HR), heart rate variability (HRV), electrodermal activity (EDA), skin conductance responses (SCRs), and the administration of the psychological stress perceived stress scale (PSS-10). Following hypnosis induction, participants were led to recall and relive a previously agreed-upon, pleasant experience, while the related psycho-physiological changes were monitored. The hypnosis session was planned on a regular working day. Physiological parameters were recorded using the Empatica E4 wristband and eSense galvanometer. Measurements were taken at baseline, during hypnosis, and after dehypnotization.

**Results:**

Participants exhibited moderate stress levels before hypnosis (mean PSS-10 = 17.1 ± 8.1). After hypnosis, a significant and large decrease in SCRs (T-test = 3.24, DF = 19, *p* = 0.002, as shown also by Cohen’s d = 0.724) and an increase in EDA (Wilcoxon = 50, DF = 18, *p* = 0.00355) were recorded, while HRV did not show significant changes. Friedman ANOVA for repeated measures models, and Nemenyi post-hoc correction indicated that the condition (basal, hypnosis, and post-hypnosis) significantly affected SCR levels (*p* = 0.00008), especially in the pre vs. post (*p* = 0.012313) and in the hypnosis vs. post comparisons (*p* = 0.00005819). Friedman ANOVA for repeated measures models and Durbin–Conover indicated that conditions (basal, hypnosis, or post-hypnosis) also influenced EDA levels, while HRV did not show any significant change (tested with ANOVA repeated measures). Pearson’s correlation showed that PSS-10 levels were inversely correlated with SCRs in hypnosis (*p* = 0.019, DF = 18, R = −0.51). A linear regression model fitting delta SCRs (post-pre physiological activation) showed that SCR-pre explained part of the outcome (R = 0.647, R^2 adj = 0.386, AIC = 99.6, *F* = 13, RMSE = 2.51, *p* = 0.002). Regarding subjective perception, 18/20 participants reported feeling better, 2 reported no difference χ^2^ = 29.2, *p* = 0.00000047, df = 2. Furthermore, the Bayesian paired samples T-test comparing SCR-pre vs. post showed a BF_+_₀ = 20.7, error % = 0.000824, which strongly indicates the superiority of the effectiveness of training in comparison to null hypotheses (>20 times probable than an effect than no effect).

**Conclusion:**

Our data suggest that hypnosis could be a valuable resource for stressed dentists. Longitudinal controlled studies and larger samples are necessary to corroborate our results and to check the durability of effects over time. Anyway, our results suggests that a short session of hypnosis (or perhaps, self-hypnosis) during a regular working day may help relieve the sympathetic stress response.

## Introduction

Dentistry is a stressful and highly demanding profession that requires strong mental and physical involvement from the dental school and may result in stress and anxiety (Collin et al., 2019; Queirolo et al., 2023; Meyerson et al., 2022). In fact, during clinical practice, dentists have to face infectious, chemical, and physical risks, as well as psychosocial stress (Moore and Brødsgaard, 2001; DiMatteo et al., 1993). According to Myers et al., the main stressors for dentists are the run behind the schedule and the management of difficult and uncooperative patients (Myers and Myers, 2004). They may, in turn, challenge psychophysical homeostasis by sudden requests that may exceed the subject’s resilience (a complex concept in medicine and psychology) and dentists’ available resources (Angeler and Allen, 2016; Facco, 2020; Angeler et al., 2018). In this regard, the Karasek–Theorell model of job-related mental strain seems valuable, but does not take into account the physical stress-related components (Karasek, 1979). In general, the literature focuses primarily on psychological aspects: for instance, Moro et al. found a 13% prevalence of burnout syndrome in dentists; while lower than in nurses and other doctors, burnout among dentists is relevant because it can worsen both dentists’ health and patients’ dental care (Moro et al., 2022). Indeed, excessive stress may affect decision-making and working performance (Chipchase et al., 2017; Plessas et al., 2018). A cross-sectional survey by Hopcraft et al. reported a current or previous self-reported depression in 26% of dentists, anxiety disorders in 23.1%, and both anxiety and depression in 6.1% (Hopcraft et al., 2023), while in the survey of Collin et al. 44% of respondents felt that work-related stress surpassed their ability to cope (Collin et al., 2019). COVID-19 pandemic contributed to worsening the working conditions of dentists as well (Owen et al., 2022).

As a whole, these disorders are associated with a high risk of maladaptive coping behavior, such as anxiolytic, antidepressant, alcohol and drug abuse, poor health, lower life expectancy, and suicidal thoughts. Many findings agree on the need for education and intervention programs to support the mental wellbeing of dental practitioners (Özarslan and Caliskan, 2021). Among these, hypnosis may help relieve anxiety and post-traumatic stress disorder as well as improve resilience (Facco, 2020; O’Toole et al., 2016; Leo et al., 2024).

According to the American Psychological Association (APA), hypnosis is “A state of consciousness involving focused attention and reduced peripheral awareness characterized by an enhanced capacity for response to suggestion” (Elkins et al., 2015). This definition, though correct, ignores the intimate psychosomatic nature of hypnosis, i.e., its capacity to modulate the somatic and neurovegetative regulation, as well as stress reactions. During hypnosis, the subject is driven to mental imagery by focused attention and absorption, resulting in so-called plastic monoideism—i.e., a powerful image perceived as if it were almost real and experienced with psychosomatic participation, a fact yielding measurable physical changes (Williamson, 2019). There is increasing evidence of the effectiveness of hypnosis for sedation and analgesia in dentistry, surgery, and invasive procedures (Tieri et al., 2023; Queirolo et al., 2024; Girón et al., 2024; Hansen et al., 2024). Actually, hypnosis can relieve dental anxiety and phobia, allowing for facing oral surgery in full wellbeing and cardiovascular stability, and is the best option in selected cases (Tefikow et al., 2013; Steenen et al., 2024; Noergaard et al., 2019; Facco et al., 2021). The effects of hypnosis in pain and psychosomatic disorders depend on the modulation of several brain areas and circuits, including prefrontal cortex, insula and anterior cingulate cortex, engendering a partial deactivation of the default mode network and an increased connectivity between the salience network, the central executive network and involved motor areas (Casiglia et al., 2020; De Benedittis, 2024; Flammer and Alladin, 2007; Flammer and Bongartz, 2003; Thompson et al., 2019; Wolf et al., 2022; Sim et al., 2024). Interestingly, besides patients, hypnosis can be potentially useful for dentists too.

The effects of hypnosis can be studied by the analysis of psychophysiological variables, such as heart rate variability (HRV), heart rate (HR), and electrodermal activity (EDA), besides validated questionnaires and fMRI (Boselli et al., 2018; Yüksel et al., 2013). These parameters may detect the activity of the autonomic nervous system, providing information about the sympatho-vagal balance, which reflects the ability to adapt to the environmental requests and, in this context, the effects of hypnosis on both the patient and the dentist (Thayer et al., 2009; Hickey et al., 2021; Koenig and Thayer, 2016; Queirolo et al., 2024; Gullett et al., 2023; Fernandez et al., 2022). EDA allows for continuous monitoring of skin electric conductance, which depends on the activity of sweat glands; therefore, it is a sympathetic index, the fluctuation of which reflects the activation or suppression of the sympathetic nervous system (SNS) (Kasos et al., 2022; Posada-Quintero and Chon, 2020; Queirolo et al., 2024). There is some evidence that hypnotic relaxation is paralleled by increased parasympathetic activity and reduced activity of SNS, a fact explaining the effectiveness of hypnosis in the treatment of conditions associated with strong SNS activation (De Benedittis, 2024; Kekecs et al., 2016; Kasos et al., 2020).

Despite an extensive literature search, we found no studies specifically investigating the relationship between hypnosis and dentists’ stress, which may be due to the general lack of high-quality studies in this area (Fisch et al., 2017; Whitehouse et al., 1996; Cardeña et al., 2013). Moreover, it is often the case that both medical doctors and dentists are more focused on caring for others than seeking care for themselves.

Hypnosis and self-hypnosis have been reported to reduce perceived stress, alleviate symptoms of burnout, and enhance overall wellbeing. Currently, only a few randomized controlled trials (RCTs) have examined the use of hypnosis to reduce occupational stress, and although the evidence is limited in quality, the findings so far are promising (Fisch et al., 2017; Whitehouse et al., 1996; Cardeña et al., 2013). Notably, some evidence has emerged suggesting that a significant number of healthcare professionals would have welcomed hypnosis as a tool for managing the stress associated with the COVID-19 pandemic (Wozniak et al., 2022). However, the practice remains rare; in one survey, only 23 out of 1,247 healthcare workers reported using hypnosis as a complementary therapy (Aveni et al., 2016). This underutilization highlights the need for further exploration in this field and serves as the rationale for conducting the present research.

This study aims to evaluate how stress management resources can be modulated by hypnosis through SNS monitoring in dentists before, during, and after an anxiolytic hypnosis session.

## Materials and methods

This prospective study was approved by the Ethical Committee of the Department of Brain and Behavioral Sciences, University of Pavia (Prot. n. [1] 131/23). The participants enrolled in this study were dentists below 45 years of age, who signed an informed consent prior to participation. Data were collected between 1 June 2024 and 31 July 2024. Hypnosis sessions were planned during a routine working day between 2 p.m. and 4 p.m. The psychological and physiological data were recorded with the following protocol:

- 3-min baseline recording of the sympathetic and parasympathetic activities with Empatica E4 wristband and eSense galvanometer;
- perceived stress evaluation by the Perceived Stress Scale (PSS-10);
- a 10-min hypnotic session, recollecting pleasant memories, during which the sympathetic and parasympathetic activities were recorded;
- a 3-min recording of sympathetic and parasympathetic activities after dehypnotization;

At the end, participants were asked “how do you feel?,” with three possible answers: equal, better, or worse.

### Exclusion criteria

Psychiatric or cardiovascular disorders (i.e., hypertension), presence of a relevant trait anxiety ≥40 evaluated with STAI-Y-2, and experience with hypnosis.

### Sample size

A sample size of 19 was suggested by *a priori* power analysis to reliably detect an effect size of *δ* ≥ 0.6 with probability greater than 0.8, assuming a one-sided criterion T-test allowing for a maximum Type I error rate of a = 0.05. Therefore, 20 participants were enrolled, also considering a possible dropout of 5%.

### Physiological variables

EDA is a property of the skin reflecting the stress-related changes in electrical conduction in response to sweat secretions. Human sweat glands have sympathetic cholinergic innervation originating from the sympathetic branch of the nervous system. There are at least three possible pathways that may lead to EDA phase alterations: (1) frontal, premotor cortex, and basal ganglion; (2) limbic system and hypothalamus; and (3) the reticular formation in the brainstem. Therefore, EDA allows monitoring of SNS activity changes before, during, and after hypnosis.

The EDA electrodes were applied on the non-dominant hand, on the distal phalanges. EDA was sampled at 4 Hz with the eSense Mindfield galvanometer and extracted as the mean value of electrodermal activity after data preprocessing, excluding 0 data points and spikes. Furthermore, a derived parameter, named skin conductance responses (SCRs), has been analyzed using the eSense algorithms. The available data in the literature suggest that a relaxed frame is between 0 and 5 SCR/min. Values in the range 5–9 indicate a mild arousal (which can contribute to increasing performance) (Boscolo et al., 2024), while higher values (in the range 10–16 SCR/min) are related to an increasing level of stress (De Benedittis, 2024; Boucsein, 2012).

HRV is a parasympathetic index reflecting central autonomic network modulation that may be measured in several different ways (Thayer and Lane, 2000; Thayer and Lane, 2009; Shaffer and Ginsberg, 2017; Beauchaine and Thayer, 2015; Thayer et al., 2012). Here, the root mean square of successive differences (RMSSDs) between normal heartbeats was chosen as the HRV index, given its high correlation with the high frequency part of the spectral analysis, which reflects vagal activity and can be calculated with a simple procedure. From photoplethysmography (PPG), inter-beat intervals (IBIs) were derived, and HRV was extracted as the root mean square of RMSSDs by calculating each successive time difference between heartbeats in ms from IBIs and then over a short-term period of 30 s.

### Perceived stress

The subjective perception of stress level was measured through the PSS-10, a test recording the perceived stress levels in the last month. Scores in the range 0–13 indicate low stress, those in the range 14–26 indicate moderate stress, and scores higher than 26 indicate high stress.

### Hypnosis

The participants were asked about previous peaceful subjective experiences. Following the induction of hypnosis, the participants were led to be absorbed in the previously agreed mental image, realizing the so-called plastic monoideism, a term introduced by James Braid in 1843 and endorsed by Franco Granone in the latter 20th century–it indicates a deep concentration in an imaginative hypnotic task engendering a powerful plastic idea perceived with psychosomatic participation—i.e., yielding both psychological and physical, measurable effects (Casiglia et al., 2019).

### Statistical analysis

Continuous data were presented as mean ± standard deviation (SD), when normally distributed, or as the median, when non-normally distributed. The Shapiro–Wilk test was used to test normality. Categorical data were summarized using absolute and relative frequencies. To compare paired data, the t-test or the Wilcoxon matched-pairs test was applied. Pearson’s or Spearman’s coefficient was used to check correlations between different parameters. The normality of residuals and homoscedasticity of variance were checked before fitting ANOVA models. A repeated measures ANOVA with condition levels (“pre,” “hypno,” “post”) was used to determine whether the primary outcomes (dependent variables: physiological variables; SCRs, EDA, and HRV) significantly differed across the three conditions. The acronym SCR-𝚫 was used to indicate the difference between data recorded at the end and the beginning of the work shift. The primary outcome will also be explored using Bayesian analysis, as it provides several advantages over traditional frequentist methods. Bayesian analysis allows for the incorporation of prior knowledge, while it offers a more intuitive understanding of the probability of hypotheses, and is particularly useful when dealing with small sample sizes, where frequentist methods may be less reliable. Furthermore, Bayesian analysis directly estimates the probability of a hypothesis, which is more interpretable in decision-making contexts. We specify that our pre-determined comparisons were SCR-pre being higher than SCR pre was higher than SCR post, and HRV in the hypnosis condition was higher compared to the other conditions. We also expected a difference according to the three conditions, with SCRs in hypnosis as well as EDA increasing from baseline.

## Results

11 women and 9 men (mean = 30, SD = 7.37) were enrolled in this study.

**Figure 1:**
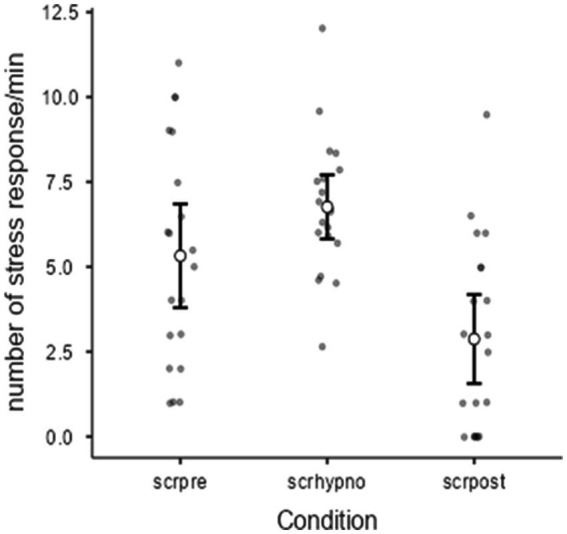
Relationship between condition (pre, hypnosis, or post) and SCRs, Friedman’s ANOVA (*p* = 0.00008). SCR-pre vs. post hypnosis in 20 subjects (T-test = 3.24, *p* = 0.002, Cohen’s d = 0.724).

As expected, the participants showed a huge decrease in sympathetic activity in the post compared to the pre-condition (t = 3.24, *p* = 0,002, Cohen’s d = 0.724) (Figure 1). While frequentist statistics focuses on the probability of the data to give a hypothesis (*p*-value), Bayesian statistics calculates the probability of a hypothesis given the data (posterior probability). This provides a more intuitive understanding of how likely a hypothesis is after seeing the data, which is often more relevant for decision-making. The Bayesian paired samples T-test showed a BF_+_₀ = 20.7, error % = 0.000824, which strongly indicates the superiority of the effectiveness of the intervention in comparison to the null hypothesis (Table 1).

**Table 1:** Aggregated data in all conditions, expressing mean, median, SD and range of the following variables: electrodermal activity (EDA), heart rate variability (HRV), heart rate (HR), skin conductance responses (SCRs), and Perceived Stress Scale score (PSS-10) in 20 participants submitted to a session of hypnosis.

Variable	Mean and SD	Median	Range
PSS-10	17.1 ± 8.1	15	[Min: 4—Max: 33]
SCR-pre	5.3 ± 3.3	5.2	[Min: 1—Max: 11]
SCR-hypno	6.8 ± 2.0	6.7	[Min: 2.6—Max: 12]
SCR-post	2.9 ± 2.8	2.8	[Min: 0—Max: 9.5]
HRV-pre	54.6 ± 21.5 ms	55.3	[Min: 21.6—Max: 99.4]
HRV-hypno	54.9 ± 12.7 ms	52.3	[Min: 31.6—Max: 79.5]
HRV-post	48.0 ± 14.9 ms	49.9	[Min: 27.5—Max: 78.2]
HR-pre	77.3 ± 10.6 bpm	75.1	[Min: 61.7—Max: 94.8]
HR-hypno	76.2 ± 8.2 bpm	74.2	[Min: 62—Max: 95.7]
HR-post	75.4 ± 7.8 bpm	73.5	[Min: 66.1—Max: 89.6]
EDA-pre	5.2 ± 2.8 µS	4.4	[Min: 2.3—Max: 12.5]
EDA-hypno	7.6 ± 4.8 µS	6.9	[Min: 1.2—Max: 21.6]
EDA-post	6.5 ± 4.4 µS	5.6	[Min: 1.1—Max: 21.1]

Pre, before hypnosis; Hypno, during hypnosis; post, after dehypnotization.

**Figure 2:**
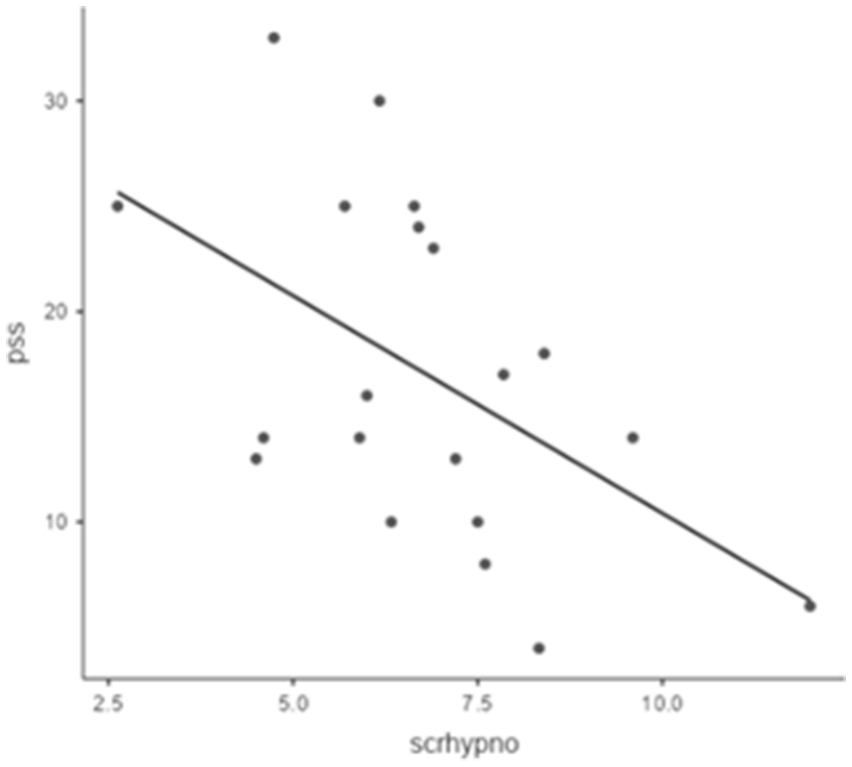
Correlation between SCR hypno and PSS-10 scale: Pearson’s (R = −0,517, DF = 18, *p* = 0.019).

Figure 1 also shows the effect of condition (pre, hypnosis, or post) on the SCR variable (χ^2^ = 18.9; *p* = 0.00008) measured with Friedman’s ANOVA (due to the violation of the homoscedasticity of variance despite the Shapiro–Wilk normality test being respected). Table 2 also reports a post-hoc comparison showing the differences between pre and post and hypno vs. post conditions. A negative moderate correlation between SCR-hypno and PSS-10 scale was found by Pearson’s test (R = −0.51, DF = 18, *p* < 0.0019) as reported in Figure 2. Although all possible correlations were searched, no other significant correlation was found.

**Table 2:** SCR Friedman’s ANOVA for repeated measures statistics and multiple comparison Nemenyi post-hoc correction.

SCR Repetead measures ANOVA (Friedman)		χ^2^ = 18.9	Acceptance: [0–5.9915], df = 2		*p* = 0.00008***
Multiple Comparison Nemenyi post-hoc correction					
Pair	Rsum difference	Q	Lower CI	Upper Ci	*p*-value
SCRpre—SCRhypno	−9	2.0125	−23.8229	5.8229	0.329
SCRpre—SCRpost	18	4.0249	3.1771	32.8229	0.01231*
SCRhypno—SCRpost	27	6.0374	12.1771	41.8229	0.00005819***

*=*p*<0.05, **=*p*<0.01, ***=*p*<0.001.

**Figure 3:**
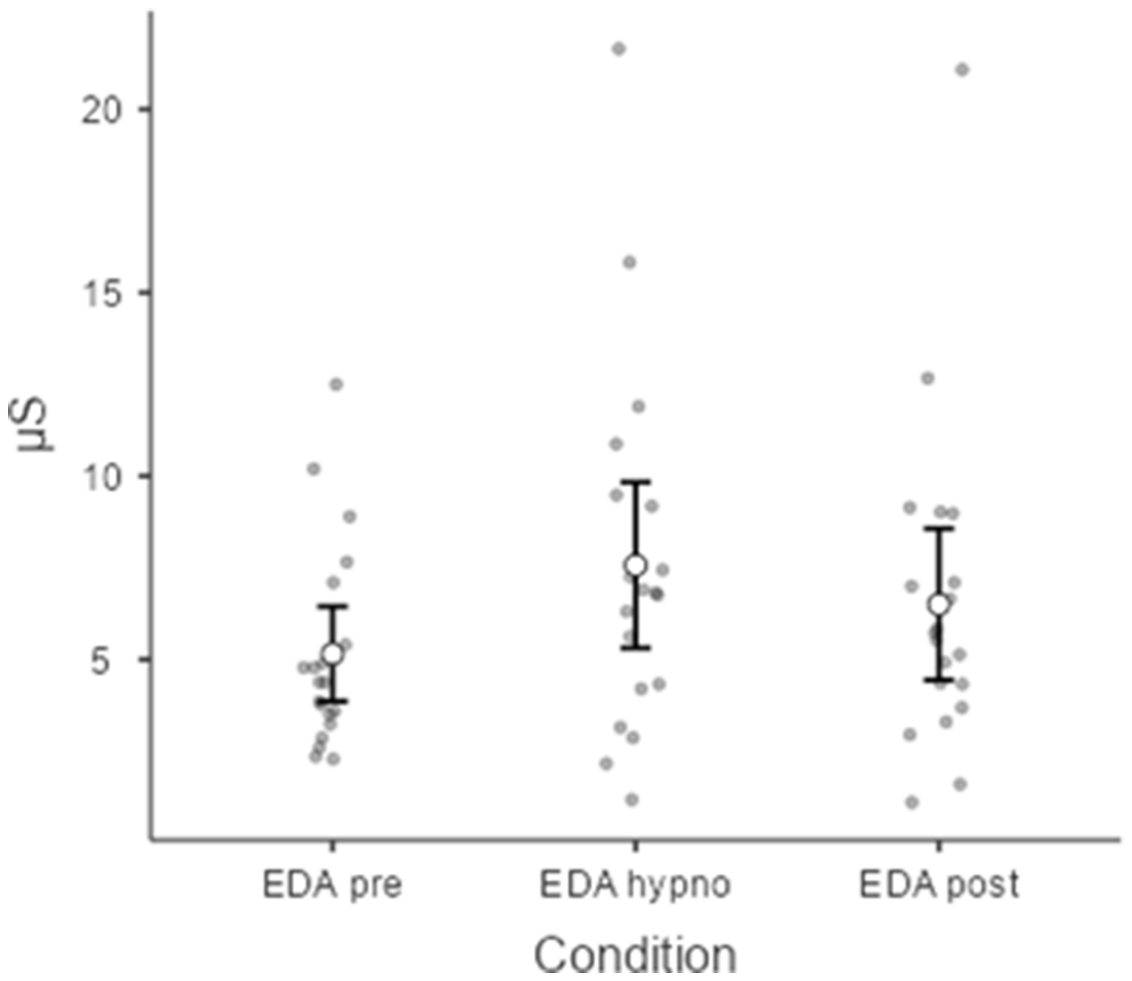
EDA before, during, and after hypnosis (Friedman’s ANOVA for repeated measurements). Chi-squared (χ^2^ = 8.40, *p* = 0.015).

EDA was not normally distributed. The analysis revealed a slight increase in EDA during hypnosis, followed by its marked decrease in the post condition (Wilcoxon = 50, DF = 18, *p* = 0.0035), while Friedman’s ANOVA was significant (*p* = 0.015). Post-hoc comparison using Durbin–Conover tests showed a difference between EDA pre vs. hypno (*p* = 0.003) and hypno vs. post (*p* = 0.044) (Figure 3). No effect of the condition (pre, hypnosis, or post) on HRV was found; however, the result of ANOVA for repeated measurements was close to the significance level (*p* = 0.054).

No effect of the condition (pre, hypnosis, or post) on HR was found.

A regression model to check if pre-hypnosis SCR value could explain the final improvement (i.e., SCR-𝚫) –explained less than 40% of the variance, R^2 = 0,386, AIC = 99.6, *F* = 13, RMSE = 2.51, *p* = 0.002. The subjective perception of stress improved in 18 out of 20 participants (χ^2^ = 29.2, *p* = 0.00000047, DF = 2).

## Discussion

Dentists, as well as other health professionals, including physicians and nurses, are subjected to relevant stress in their profession. In our sample, dentists’ stress levels were mild in basal conditions, both on a physiological (SCR-pre = 5.3) and a psychological level (PSS-10 = 17.6); these data confirm the previous literature about dentists’ stress as well as the positive effect of hypnosis in decreasing it (Myers and Myers, 2004; O’Toole et al., 2016; Cooper et al., 1988). The physiological component of the effects of hypnosis, i.e., the SCR decrease, has turned out to be even larger than the predicted one in the *a priori* power analysis. Similarly, the sample size was overestimated because the effect Cohen’s d = 0.724 was larger than the predicted one, 0.6, i.e., even a smaller sample size could have reached the minimum threshold to show a significant effect.

To our knowledge, no similar studies have been published so far on the effects of hypnosis on dentists’ stress. Interestingly, our data show an increased sympathetic tonic component associated with a decreased phasic component, i.e., an increase of approximately 25% of the former and a decrease of approximately 50% of the latter. This ostensible contradiction (i.e., the opposite deviation of two indexes of sympathetic activity) may arguably be explained in terms of challenge vs. threat. The increased sympathetic response may result from both challenge and threat, the former not being negative. Challenge is marked by an adaptive response (e.g., sports competition) where personal resources are superior or equal to the demands, while threat engenders a maladaptive approach entailing an imbalance between them. As a result, successful challenges are associated with increased catecholamine release and related increase in HR, cardiac output, and decreased total peripheral resistance, in turn rearranging the distribution of blood flow to allow for improved circulation in the brain, heart, and muscles. Enduring threats activate the pituitary-adrenocortical axis besides the medullo-surrenal component, resulting in increased peripheral resistance and decreased cardiac output, a fact leading to worse performance. Furthermore, the subject’s sympathetic activation depends on the subject’s motivation and how the task is perceived (i.e., as a challenge or threat) prior to performance, allowing at least partly to predict it.

According to Blascovich and Tomaka (Shaffer and Ginsberg, 2017), an increase in tonic sympathetic activity (as defined by EDA) may occur in both challenge and threat. EDA has two components—a slow-changing tonic one –dependent on environmental conditions (such as temperature and humidity) as well as individual level of arousal—and a phasic one (SCR); the latter is fast-changing and reflects the activation of the sympathetic nervous system in response to several events, including emotional stimulation and related attentional load. The scientific literature mostly relies on EDA, but with some new promising algorithms, it is possible to deconvolute the raw EDA signal to better recognize the small peaks of the fast component. It is reasonable to argue that the phasic component may be more sensitive to challenge and transient threatening stimuli—a fact allowing it to immediately fit with changing events in real life—while the tonic one is more relevant in hardships, especially when perceived as enduring threats. If this is the case, life’s adversities can be interpreted as challenges or threats according to both environmental conditions and individual standpoint and resilience, including the capacity to improve it (Facco, 2020). Given the different speed response of the two components, the tonic one seems to be less suitable than the phasic one for an immediate reaction to stimuli.

Unlike other reports (De Benedittis, 2024; Yüksel et al., 2013), in our study, no HRV increase was found; a possible explanation is that HRV can be estimated by several indexes—e.g., SDNN, PNN50, lambda 25, LF, and HF (Hautala et al., 2010; Tulppo et al., 2005; Hayano et al., 2011; Shaffer and Ginsberg, 2017)—the meaning of which is still uncertain (e.g., the LF/HF ratio) and does not seem to closely reflect the sympathovagal balance (Billman, 2013; Rahman et al., 2011; Martelli et al., 2014).

In a previous study, 91% of dentists were stressed by anxious patients, while, unsurprisingly, hypnosis was not used in their clinical practice to manage patients and/or as self-hypnosis to relieve stress. Accordingly, only 2% of general dentists practice hypnosis, though it has proved to be time and cost-effective in invasive procedures, surgery, and in dentistry, allowing for effective management of patients’ anxiety and phobia in a few minutes (Facco et al., 2021; Rosendahl et al., 2023; Mcknight-Hanes et al., 1993; Burghardt et al., 2018). In our sample, even a single 10-min session proved to be enough to relieve dentists’ stress during a routine working day, suggesting that it could also be implemented as self-hypnosis (Hill et al., 2008).

The main limitations of this study are the small sample size and the only evaluation of the immediate effects of a single session of hypnosis. Second, no control group was used, and although we adopted a within-subject pre-post design in which each participant served as their control, the absence of an active or inactive control group makes it difficult to draw causal conclusions. Nevertheless, these findings offer preliminary insights into the potential mechanisms by which hypnosis may influence stress regulation in real-life professional settings and provide a rationale for future confirmatory studies using larger samples and randomized controlled designs. The aforementioned limitations, however, are balanced by a Bayesian Factor estimate >20, with a very small error associated with the measure. This result can help mitigate the limits of frequentist tests on small sample sizes, decreasing Type I and Type II errors. This is a strong result according to the Jeffreys scale, where a value >10 indicates strong evidence for the alternative hypothesis, i.e., it is 20 times more probable that there is an effect of treatment than there is no effect (Jeffreys, 1961). Further studies on larger samples are required to check: a) the relationship between psychological, neurovegetative, and neurophysiological data (e.g., EEG and event-related potentials; Rosendahl et al., 2023; Mcknight-Hanes et al., 1993; Burghardt et al., 2018). The pre-post design of this study has not explored the duration of the effects of hypnosis over time, but suggests that a 10-min session of hypnosis may positively affect the psychophysical regulation of dentists. Further studies using more than one session or self-hypnosis are required to check whether hypnosis may allow for steady improvements and to evaluate possible effects on executive functions and resiliency.

## Data Availability

The raw data supporting the conclusions of this article will be made available by the authors, without undue reservation.
